# New Possibilities in Life with Type 2 Diabetes: Experiences from Participating in a Guided Self-Determination Programme in General Practice

**DOI:** 10.1155/2018/6137628

**Published:** 2018-03-20

**Authors:** Bjørg Karlsen, Bettina Rasmussen Bruun, Bjørg Oftedal

**Affiliations:** ^1^Faculty of Health Science, University of Stavanger, 4036 Stavanger, Norway; ^2^Department of Home Care, Municipality of Stavanger, Stavanger, Norway

## Abstract

Research suggests that guided self-determination programmes can support self-management of diabetes by empowering self-determined goal setting and competence building. As most research in this area has focused on people with type 1 diabetes, knowledge is lacking on how adults with type 2 diabetes mellitus experience participation in such programmes. This study reports the modelling phase of a complex intervention design that explored the experiences of adults with type 2 diabetes who participated in a nurse-led guided self-determination programme in general practice and examines how the programme affected patients' motivation to self-manage diabetes. The qualitative design with semistructured interviews included 9 adults with type 2 diabetes who participated in the programme. Qualitative content analysis was used to analyse the data. The findings indicate that the participants experienced new life possibilities after participating in the programme, which seemed to have a positive influence on their motivation for self-management. Through reflections about how to live with diabetes, the participants reinterpreted their life with diabetes by gradually developing a closer relationship with the disease, moving towards acceptance. The fact that dialogue with the nurses was seen to be on an equal footing helped support the participants to become more self-determined.

## 1. Introduction

In this paper, we report on the modelling phase of a complex intervention design aimed at exploring the experiences of adults with type 2 diabetes (T2DM) who took part in a nurse-led guided self-determination (GSD) programme in general practice.

An alarming increase in the prevalence of T2DM is evident worldwide and it is a growing public health problem in Norway [1]. T2DM is a demanding chronic disease for the individual, who faces many challenges in taking responsibility for the disease. There is no cure and people with T2DM are recommended to engage in multiple self-management behaviours to achieve adequate blood glucose control and prevent long-term complications [2]. Diabetes self-management involves an active and ongoing process including behaviours such as healthy eating, regular physical activity, blood glucose monitoring, and medication in addition to problem solving and coping [2]. However, for many people with T2DM appropriate self-management is difficult to achieve [3]. Research suggests that people with T2DM find the attainment of treatment goals challenging and many fail to reach optimal treatment outcomes with regard to glycosylated haemoglobin (HbA_1C_), cholesterol, and blood pressure [4]. Support from health care professionals is required for patients to implement the majority of self-management tasks in daily life [5, 6].

In most previous reviews, educational and behavioural interventions for promoting self-management among adults with T2DM have demonstrated only moderate effects [3, 7]. However, one review of group-based diabetes self-management education has shown improvements in clinical, lifestyle, and psychosocial outcomes [8]. In addition, a recent review by Pillay et al. [9] has suggested that support components should be included when training people to self-manage their disease. Thus, ongoing educational, behavioural, and clinical support is needed to sustain changes made during diabetes self-management education.

Given the increasing prevalence of T2DM and the somewhat ambiguous research results, there is still a need for ongoing educational and behavioural support to deliver self-management support interventions for people with T2DM. As a result, we modified a GSD programme for use by adults with T2DM [10]. The GSD programme aims to support diabetes self-management by empowering self-determined goals and competence building among people who have difficulties in diabetes management [11]. It has a theoretical foundation in self-determination theory (SDT) where three basic psychological needs (autonomy, relatedness, and competence) are central [11, 12]. The theory stresses the significance of having a sense of volition and choice in one's self-management and goal setting, of feeling supported and related to others, as well as feeling that one can achieve self-management behaviours. Through an internalization process, people try to accomplish external demands, norms, and values by integrating them and thus perceiving them to be internal or autonomous [13]. The theory also emphasises that autonomous self-regulation and perceived competence for healthy behaviour engagement can be facilitated by autonomy support [13–15]. Autonomy-supportive approach among health providers is therefore crucial in order to support patients' self-engagement for change, which is central for the development and maintenance of motivation [14, 16, 17].

SDT has already been applied to health care and health behaviour change [14–17] as well as to T2DM management [18, 19]. In diabetes, research has indicated the effectiveness of GSD in group training among both younger women and adults with type 1 diabetes [20, 21]. Previous research also suggests that GSD can be adapted to other patient groups in other contexts [22, 23].

To our knowledge, the GSD approach has not yet been studied from the perspective of adults with T2DM. Accordingly, it is timely to consider how a GSD programme might promote motivation for self-management among adults with T2DM. Therefore, the aim of this study was to explore the experiences of adults with T2DM who took part in a GSD intervention in general practice and to assess how this influenced their motivation to self-manage diabetes.

## 2. Methods

### 2.1. Design

This study is based on the findings from the modelling phase of a complex intervention design built on the UK Medical Research Council framework [10, 24]. The intention of the modelling phase was to provide important information in advance about how to design a full-scale intervention [24]. To provide this information, we used a descriptive and explorative qualitative approach. Data were collected by means of individual interviews and the study was conducted in four general practices in the southwestern part of Norway.

### 2.2. Guided Self-Determination Programme Gradually Adjusted for Adults with Type 2 Diabetes Mellitus

In Norway, T2DM care is primarily delivered by general practitioners and registered nurses in general practice. Thus, general practice was selected as a suitable intervention site for this study. Four nurses experienced in diabetes care from different practices were invited to attend a structured and supervised theoretical and practical training programme in GSD to develop their counselling skills in nurse-led consultations for patients with T2DM [10]. The practical training was delivered to patients in their own practice to supplement regular care, which consists of an annual consultation at the general practice. This consultation focuses on complications, treatment goals, and psychosocial factors. In addition, patients are recommended to obtain measurements of HbA_1C_ every three to four months or on an individually agreed basis [25].

To guide the T2DM patients towards setting autonomous goals and active planning for effective diabetes self-management, the GSD programme, originally developed for people with type 1 diabetes [26], was evaluated and gradually adjusted for patients with T2DM during the modelling phase [10]. The number of consultations was reduced from seven to four to make it more time efficient and the number of semistructured reflection sheets used at the consultations was reduced from 21 to 13 in accordance with the needs of people with T2DM. This reduction was done without losing essential elements in the reflection sheets covering the patient-provider relationship, life with diabetes, the relationship between the ideal and reality, and change. The reflection sheets were retained to support people in prioritising problems and to prompt self-determined goals [10]. In addition, it was important to follow general GSD principles about establishing an optimal collaboration between nurses and patients, in which patients clarify their values and express their needs [11]. More details about adjusting the GSD programme to patients with T2DM are presented in Karlsen et al. [10]. Prior to the consultations, the patients prepared the sheets at home by recording their reflections. The themes addressed on the reflection sheets were then discussed with the nurse at the consultations with the intention of stimulating the patients to become active in the change process.

### 2.3. Recruitment

As part of the training programme described above, the four nurses working in different general practices invited a total of 16 adults with T2DM (four patients each) to participate in the nurse-led GSD programme and to be interviewed individually after completing the programme. Criteria for inclusion were as follows: (1) having a T2DM diagnosis; (2) being aged > 18 years; (3) having a disease duration > 3 months; and (4) being able to communicate in Norwegian. Half of those invited took part in the original version of the GSD programme (with seven consultations), whereas the other half participated in the adjusted version of the programme [10]. To obtain a balanced picture of how adults with T2DM experienced their participation in the nurse-led GSD programme, we collected data from patients in both groups to get an adequate number of informants. We assumed that the different experiences of the participants from the two groups would be valuable for the aims of the study. Those who participated in the original programme version were also interviewed about suggested programme adjustments for adults with T2DM. Reactions to these suggestions will be reported elsewhere. Of the 16 patients invited to participate, 9 agreed to be interviewed after completing the intervention. Of these, four participated in the original GSD programme and five took part in the adjusted programme. The 9 participants comprised 5 men and 4 women aged between 36 and 67 years, with a disease duration ranging from 2 to 15 years.

### 2.4. Data Collection

The interviews were carried out in 2015, two weeks after the participants completed the programme. The patients chose where to be interviewed: at their homes, at their work place or in a coffee bar. Due to practical considerations, one interview was conducted by telephone. The second author (BRB) performed all interviews according to a semistructured interview guide developed for this study. The interviews began with an open question: Could you please tell me about your experience with the GSD programme? Supplementary questions were asked to invite clarification and explanation about variations in the participants' experiences of the GSD programme content, self-management, motivation, relationships with nurses, family, and friends, and change work. The interviews lasted from 40 to 60 minutes except for the telephone interview, which lasted 15 minutes. They were all audiotaped and subsequently transcribed verbatim.

### 2.5. Data Analysis

The transcribed interviews were subjected to qualitative content analysis as described by Graneheim & Lundman [27]. The analysis was carried out in several stages to highlight both the manifest and latent content. Initially, the first author (BRB) read and reread the transcribed text several times to gain an understanding of its overall content. Meaning units corresponding to the aim of the study were identified and condensed while preserving the core. The condensed meaning units were labelled with codes, which were compared for similarities and differences before being consolidated into subthemes. The next step was to analyse the subthemes on a more abstract and interpretive level resulting in two themes relating to how the participants perceived variations in their experiences of self-management, motivation, relationships, and change work. Finally, one overall theme that described the subthemes and themes were identified. In order to ensure the credibility of the analysis, the research group discussed and revised the subthemes, themes, and overall theme several times until consensus was obtained.

### 2.6. Ethical Considerations

The Norwegian Regional Committee for Medical and Health Research Ethics (REK west number 2015/60) approved the study. Verbal and written consent was obtained prior to all interviews and the informants were guaranteed anonymity as well as the right to withdraw from the study at any time.

## 3. Results

The analysis identified one overall theme relating to the participants' experiences in the nurse-led GSD training intervention:* new possibilities in life with T2DM. *This theme reflects the following two underlying themes: (1) a reinterpretation of living with diabetes and (2) a motivating dialogue on an equal footing, together with their corresponding subthemes.  Table 1 gives an overview of the overall theme together with the two underlying themes and their corresponding subthemes.

The two themes, each of which was based on two subthemes are presented in more detail below. Quotations are included for each subtheme to illustrate the meaning of the text and to help clarify interpretation.

### 3.1. A Reinterpretation of Living with Diabetes

This theme emerged from the participants' descriptions of how GSD counselling had initiated processes that led to a reinterpretation of living with diabetes, as illustrated by the following two subthemes: (1) inviting diabetes into one's life and (2) finding new resources and possibilities in the disease.

*Inviting Diabetes into One's Life*. The majority of the participants reported that the counselling had resulted in increased awareness of having diabetes, making them ready to integrate diabetes into their lives. One participant said that through the conversations with the nurse he gradually came to terms with the fact that he had diabetes and had to deal with it seriously:

It was those working at the GP's office who told me that I have type 2 diabetes. Even then – yes, okay. I hear what you are telling me, but I am not sure that I am quite there. However, this is what has been so great about these conversations with the nurses. For yes, I realise now that I have it (diabetes). After these conversations, I take it very seriously.

Another participant reported that little by little he came to understand that he had not really dealt with the disease in a serious way before. Instead, he had ignored it and put it aside:

The most meaningful is probably that I was confronted with my own previous opinions – I had really excluded it. I have been taking my medicine, I have been doing what I have been told, but all the same refused to relate to the fact that I have this disease.

Some participants also emphasised that it was better to reflect on how to deal with the disease in a serious way than to feel guilty about making bad choices, as illustrated in the next quotation:

It is a bit more serious when you start to reflect on the disease. That you really have a disease that you most likely must have for the rest of your life. Therefore, it will be how you then choose to deal with it. It is more useful, instead of just walking around, thinking: You should have gone for a walk today, now you have eaten an extra piece of cake, right?

The majority of the participants said that reflections about “Your life with diabetes” and “Focus on change,” which highlighted how much time and space diabetes takes in daily life, were helpful:

It was really a bit of this, the place diabetes has in my life. To be challenged on what space diabetes takes in my life. It was quite useful. Because, as I said, that is probably one of my main problems. I do not let it fit into my life. I just ignore it.

These reflections made them aware how much attention they gave to the disease and how attentive they should be in the future, as expressed by this participant:

It means that through conscious choices, diabetes will take less space…. (…) Life is more controlled by habits, good habits, true? Then the diabetes will take less space, because it is in a way treated better.

Most of the participants expressed satisfaction about working with the reflection sheets, which for many had resulted in intuitive reflections and thoughts and a closer relationship with diabetes. On the other hand, a few expressed dissatisfaction because they perceived the GSD counselling as inappropriate and not suitable for them. They claimed to have no problems with the disease and felt they should not have been included in the target group sample.

*Finding New Resources in the Disease*. Several participants said that taking part in the counselling programme inspired them to make their own decisions and find alternative ways to handle problems in everyday life. Some found it valuable to be empowered to reflect on diabetes-related challenges and to find their own ways of solving them, as illustrated in the following quotation:

Yes, this was the good thing with the questions there. So you are forced to sit down and then: Okay. What is troubling me and why do I have the problems and what can I do with the problems? In addition, you must find the answers yourself and so on.

Some participants argued that the counselling had resulted in their viewing diabetes in a more positive way and in an increased awareness of finding possibilities and resources through the disease. One participant said:

Nevertheless, what I realised about this was that I could even consider the disease as a kind of resource in my life. I can use it as something positive and reflect like this, that I now, in a way, have a sort of supervisor built into me telling me what is good and what is bad.

Several participants found that they reached a conscious turning point in how to think about diabetes during the GSD programme. They said they had reformulated diabetes from being an enemy to being a friend, as expressed in the following quotation:

And thus I can use it as an asset, rather than…. Then [I] look at it like some kind of a friend, if you can say it like this, instead of an enemy. And I feel that I reached this when I worked with it. That it became a sort of an awakening for me.

One participant suggested that he had changed from being ashamed of having diabetes to looking at the disease in a more positive way:

In a way, I discovered the disease as a resource and could use it as something good instead of something negative in life. It is a big thing for me that this has emerged, instead of walking around being ashamed.

The majority of the participants valued the counselling as an important tool to help them mobilise resources and let their will power control their lives instead of the disease. Some spoke of the benefits of including family members as a resource. Others said that they had learnt to ask for support from colleagues when implementing new exercise habits and had invited them to join their daily exercise. In addition, working with the reflection sheets was useful for many participants over time. The reflection process accompanied them in their daily routines not only when working with the sheets and talking with the nurses. Even after completing the counselling programme, some claimed that they frequently looked back at the sheets to maintain motivation and to bring new possibilities into focus.

### 3.2. A Motivating Dialogue on an Equal Footing

This second theme describes the participants' experiences of the dialogue with the nurses, which they perceived as motivating and on an equal footing. It is illustrated by the following two subthemes: (1) being seen and heard and (2) becoming self-determined as a result of support.

*Being Seen and Heard*. Several of the participants spoke about previous experiences with the health care system where they were met in an impersonal way, often with a focus on the clinical aspects alone. All the participants, however, expressed satisfaction with the relationship with the nurses during the counselling process, which they considered as nonjudging and positive, as exemplified in the following quotation:

It is not judging. Had it just been negative: now you have to… then I do not think I would have found the motivation. There is always something positive in it even if there is something negative.

The dialogue with the nurses was described with words like “co-player,” “motivator,” “conversation partner,” and “external support.” Many participants described the nurses' feedback and support as a source of motivation, support, and appreciation:

I still notice with this method here, it became a completely different dialogue. It became much more positive, that was one side. I noticed that very well. In addition, you get appreciation, support on having written down some wise thoughts.

Some participants, who had already started to become more goal-oriented in order to change their habits and were therefore motivated even before taking part in the intervention, stated that their motivation was further strengthened. This was more because of the relationship with the nurses than the actual counselling method. Getting specific information and advice about diet was mentioned as especially useful for many. Previously, they had been taught about what to eat but had challenges in transforming general advice about diet into concrete guidelines for use in daily life. However, in this intervention, the nurses had provided individual and tailor-made feedback to the participants: “It is through her I have become certain of: Yes, you can eat that.”

Having in-depth conversations with the nurses was considered to be positive. Many participants described the dialogue with the nurse as a joint exchange of thoughts and experiences, which resulted in new knowledge and helped the learning process. In addition, participants valued the fact that the dialogue focused on teamwork rather than providing information only, as expressed in the next quotations:

When I finished the process, she was very pleased. We had talked and a lot of things were brought out into the open and we had discussed the goals. So, we ended in a very good place, and we felt we had done a good job.

Having a person who I am familiar with and who listens to me and gives constructive feedback to what I say. That is important.

*Becoming Self-Determined as a Result of Support*. All the participants were clear about their responsibility to self-manage the disease and follow-up treatment. They had become more aware of this responsibility through the counselling and had started to work towards reaching the goals set in the GSD programme:

Now, I am quite aware of it, much more certain. It is up to me. There are many things that have become much clearer to me during these conversations, I think. So I have gained much from it.

Well, it is me who controls everything, I also felt this when I took part in this. That it was my choices and my inputs, which I myself put on the sheets and that we discussed.

Although the participants were aware of making their own decisions, they also claimed that the decisions should be made in collaboration with doctors and nurses and that, in this way, the responsibility would not rest only with the individual. It was important for them that the decisions were made with support and counselling from a professional. One of the participants said:

It is me who is in charge and the health care professionals are on the side line. So it is my responsibility, but with the health professionals on the side line, counselling, asking follow-up questions and, in that way, being able to guide and correct a little, if there should be a need for them.

Several participants also reported that they became more committed to taking responsibility for their diabetes. They felt responsible when enrolling in the GSD programme. But this was reinforced by the knowledge that the nurses were also investing time and effort on them:

That is what I think and that some people might be like me, that being committed to a programme, saying yes to attend a programme, you will follow it up in a completely different way than if you are sitting alone with all [the] responsibility for your own health.

For some participants, the counselling resulted in greater will power and a stronger feeling of determination towards reaching the goal, as illustrated in the following quotation:

I am even more aware that I must exercise and, in the worst periods, it has not been like that. Now it is not a question anymore. I have to.

All the participants expressed satisfaction with the nurses' counselling over time and many felt that they had developed a constructive relationship with them. However, this experience differed depending on the number of consultations. Among those attending the original version of the programme, some claimed that seven consultations were too many, whereas others found the number appropriate. Those participating in the adjusted programme version (with four consultations) also expressed some variations in experience. However, independent of the number of the conversations, all participants claimed that it was important to have ongoing dialogues with the nurses when working towards goals for change and maintaining motivation. It was vital to have a competent nurse to discuss different challenges with. They were glad to have one nurse to rely on who was looking after them.

## 4. Discussion

The present findings provide new insight into the experiences of adults with T2DM who participated in a nurse-led GSD training programme in general practice. Our findings indicate that the GSD programme changed the way people with T2DM reflected on diabetes and self-management. Through the counselling process, many of the participants experienced a reinterpretation of living with diabetes and a motivating dialogue with the nurses on an equal footing, which resulted in seeing new possibilities in life with diabetes. In the next section, we will discuss these findings in relation to earlier research and to the central tenets of SDT.

### 4.1. A Reinterpretation of Living with Diabetes

Our findings suggest that the GSD counselling started a process among the participants leading to a reinterpretation of living with diabetes. They indicated the importance of being aware of having the disease and thus gradually* inviting diabetes into life*. This adds to findings in a previous study suggesting that GSD counselling for adults with type 1 diabetes resulted in greater acceptance of diabetes and thus a realisation of the need to deal with the disease [28].

Only a few of the participants in the present study had accepted the disease before they took part in the GSD programme; however, the majority reached such an awareness during the counselling process. The absence of this awareness before attending GSD counselling could reflect the fact that T2DM initially presents itself as a disease with mild symptoms and which does not necessarily influence people's daily life. This could have weakened the motivation to make changes and self-manage the disease. According to SDT, lack of awareness, recognition and acceptance can hamper motivation to change as long as people are not aware of the importance of doing this [29]. After taking part in the programme, however, the participants said that they found satisfaction in working with the reflection sheets, which for many resulted in intuitive considerations and thoughts and a closer relationship with diabetes. Some participants even had to acknowledge that they had the disease. Our findings indicate that the participants changed their relationship with diabetes during the counselling period from not having any relationship at all to a closer relationship. Instead of hiding and ignoring the disease and feeling guilty about wrong choices, they gradually perceived the benefits relating to diabetes in a more serious way. These findings of developing a closer relationship with diabetes together with less self-blame could suggest that counselling activates the process of internalization where participants try to incorporate the external influence of the counselling to more internal and autonomous regulation behaviour [30].

In addition, the findings indicate that the counselling initiated a reflection process among the participants about how much time and space diabetes does and should take up in daily life. For many, this process led to increased awareness of the need to give diabetes enough space to enable them to self-manage the disease satisfactorily, reflecting increased motivation. Our findings are in line with earlier intervention research demonstrating increased awareness of the disease and new ways to consider how to self-manage [31, 32].

In contrast to these findings, some participants found the counselling irrelevant as they did not have any problems living with diabetes and felt that they did not belong to the target group sample. This relates to the discussion of consequences for motivation when an activity is not perceived as valuable enough and could indicate that the GSD programme is not appropriate for all participants. The original GSD programme was developed for people with type 1 diabetes who were struggling with the disease; such a recruitment strategy was not part of this study. Thus, in order to recruit eligible participants who want to take part in and would value such an intervention, it seems important to provide appropriate inclusion criteria.

In addition to inviting diabetes into life, the findings also suggest that the participants found new* resources and possibilities *by viewing diabetes as a friend rather than an enemy. This is an interesting finding indicating that GSD counselling has the potential to transform negative views of the disease into positive ones. Many participants felt that transferring the focus from the threat of medication and late complications to using resources and possibilities increased motivation. This reflects important aspects of empowerment that characterise a positive focus on resources instead of compliance with treatment demands [33]. According to Deci & Ryan [30], it is important to strengthen the internalization process by moving the focus from external control such as compliance to looking for possible resources. In addition, the findings indicate that the participants had become aware of the kind of resources they could rely on in their social network. Some had started to involve family members and colleagues in their process of making changes and had asked them for support by inviting them to do exercise together. This reflects the need for relatedness or belongingness with the possibility of cohering with the group, to feel connection and caring. According to SDT, such relatedness may provide a motivational basis for internalization, ensuring effective transmission from the group knowledge to the individual and a more cohesive social organisation [30]. Earlier research has shown that group education focusing on lifestyles resulted in increased ability to mobilise support from others [34]. Although our study focused on individual counselling and therefore did not include the concept of support from other group members, the present findings indicate that individual counselling also has the potential to strengthen the ability to mobilise support. Findings from the present study suggest that the participants perceived their conversations with the nurses as an ongoing source of support even after completion of the counselling, helping them to keep up focus and motivation in order to make good choices for self-management in everyday life. This may reflect that the counselling had a long-lasting influence as a positive external motivation and resource even after completion of the programme.

### 4.2. A Motivating Dialogue on an Equal Footing

The findings indicate the importance of being motivated by the dialogue with the nurse on an equal footing. The dialogue was experienced as safe and positive and gave the participants a feeling of* being seen and heard*. This adds to the findings of a previous study by Zoffmann and Kirkevold [28] demonstrating that people with type 1 diabetes experienced a reciprocal you-I relation through the GSD counselling, leading to a positive, nonjudging and supportive atmosphere. Another interesting finding in our study suggests that the participants were motivated by receiving constructive feedback on thoughts and reflections in the counselling process. According to Deci & Ryan [30], the experience of competence can be strengthened through positive feedback. Our findings could thus underline the importance of the nurses' feedback as a positive external influence, reflecting promotion of more internal and autonomous regulation being internalized.

Our findings also indicate that receiving tailored advice about diabetes management, such as a healthy diet, helped the participants to transform general knowledge to more practical use. The patients emphasised how the nurses used their own knowledge and experiences in the dialogue to tailor this to the individual patient, who in this way learnt to make his or her own decisions. According to Anderson & Funnell [33], stimulating empowerment is a basic feature in patient-centred care. Through equal dialogue based on the nurses' expert knowledge on diabetes and the patients' expert knowledge on daily life, the patients were empowered to make their own decisions about self-management. This is also reflected in our findings. The participants claimed to have done good work together with the nurses and were motivated to continue the working process with self-management.

Our findings indicate that the participants had* become self-determined by support*. The participants claimed they were involved in decisions about treatment and self-management, reflecting the fact that GSD counselling stimulated vital aspects of empowerment, such as involvement and shared decision making [35]. This is also in line with the study by Zoffmann and Kirkevold [28] showing that GSD for adults with type 1 diabetes stimulated shared decision making between patient and nurse. Moser et al. [36] demonstrated in their study that shared decision making is a central component in stimulating the autonomy of patients. According to SDT, shared decision making can be considered as an autonomous activity as long as the person has accepted it [13, 30]. This points to our findings that many patients accepted making shared decisions in cooperation with the nurses. They considered this to be more positive than sitting alone and taking full responsibility themselves.

Although many participants were clear about their own responsibility for self-management even before taking part in the GSD programme, our findings indicate that awareness of self-determination and own responsibility for self-management increased through the counselling. Being aware of taking responsibility is central in both SDT [30] and the concept of empowerment [33]. According to SDT, awareness is an important step forward towards a more internal and autonomous behaviour [30].

Our findings also suggest that the participants emphasised the importance of being supported by the nurses on an ongoing basis in order to carry out goal-oriented work and get support, particularly in periods when struggling with diabetes-related challenges. This points to the importance of the nurses' role in general practice and the significance of being followed up over time, which is also highlighted in previous studies [37, 38].

### 4.3. Methodological Considerations

It is possible that the credibility of this study was affected by the fact that we did not give the participants the opportunity to check our interpretation of the findings presented in the subthemes. However, it should be noted that the subthemes in Results section are generally illustrated by the rich use of quotations, which should have enhanced transparency. In addition, to reinforce the credibility of the data collection, the same researcher (BRB) conducted all the interviews. A group of researchers also discussed the findings and interpretations, which should have reinforced credibility. The dependability of the research was ensured through use of the same interview guide with each participant. In addition, the interviews were audiotaped and transcribed verbatim. The transferability of the findings to another context was enhanced through accurate descriptions of appropriate quotations as well as the whole research process.

## 5. Conclusion

This study is one of few to provide qualitative insights into how adults with T2DM experience taking part in a nurse-led GSD training programme. Our findings indicate that the participants experienced new possibilities in life with T2DM after taking part in GSD counselling, which in turn seemed to have a positive influence on their motivation for self-management. Through reflections about how to live with diabetes, the participants reinterpreted their life with diabetes by gradually developing a closer relationship with the disease, moving towards acceptance. In addition, they reported seeing diabetes more as friend than an enemy and, in this way, discovered new resources in the disease. Another finding indicates that the participants perceived that dialogue with the nurses on an equal footing was a motivating factor, which acknowledged them, supporting them continuously in becoming more self-determined. Nurses involved in T2DM care in general practice should be aware of providing a safe and ongoing tailored dialogue based on the GSD programme. Our findings also point to the importance of supporting people with T2DM by strengthening their self-determination through reflections and goal orientation and thus transferring external influence to inner motivation.

The findings from this modelling study may serve as a basis for future research aimed at broadening our understanding of the dynamics of participating in a nurse-led GSD training programme and provide important information for the development of a full-scale intervention evaluation.

## Figures and Tables

**Table 1:** Overview of overall theme, themes and subthemes in the qualitative content analysis.

Overall theme	
New possibilities in living with type 2 diabetes	
Themes	Subthemes
A reinterpretation of living with diabetes	(i) Inviting diabetes into life
	(ii) Finding new resources in the disease
A motivating dialogue on an equal footing	(i) Being seen and heard
	(ii) Becoming self-determined as a result of support

## References

[1] Atlas I. D. (2015). *International Diabetes Federation*.

[2] Funnell M. M. (2007). National standards for diabetes self-management education. *Diabetes Care*.

[3] Heinrich E., Schaper N. C., de Vries N. K. (2010). Self-management interventions for type 2 diabetes: a systematic review. *European Diabetes Nursing*.

[4] Mostert Wentzel K., Nel C., Van Rooijen A. (2008). High levels of self-efficacy in patients with type 2 diabetes attending a tertiary level clinic. *South African Journal of Physiotherapy*.

[5] Boström E. (2013). *Proximity and distance. Challenges in person-centred care for diabetes specialist nurses in primary healthcare in Department of Nursing*.

[6] Powers M. A., Bardsley J., Cypress M. (2015). Diabetes self-management education and support in type 2 Diabetes: a joint position statement of the American Diabetes Association, the American Association of Diabetes Educators, and the Academy of Nutrition and Dietetics. *The Diabetes Educator*.

[7] Radhakrishnan K. (2012). The efficacy of tailored interventions for self-management outcomes of type 2 diabetes, hypertension or heart disease: a systematic review. *Journal of Advanced Nursing*.

[8] Steinsbekk A., Rygg L. O., Lisulo M., Rise M. B., Fretheim A. (2012). Group based diabetes self-management education compared to routine treatment for people with type 2 diabetes mellitus. A systematic review with meta-analysis. *BMC Health Services Research*.

[9] Pillay J., Armstrong M. J., Butalia S. (2015). Behavioral programs for type 2 diabetes mellitus: A systematic review and network meta-Analysis. *Annals of Internal Medicine*.

[10] Karlsen B., Oftedal B., Lie S. S. (2016). Assessment of a web-based Guided Self-Determination intervention for adults with type 2 diabetes in general practice: A study protocol. *BMJ Open*.

[11] Zoffmann V., Hörnsten Å., Storbækken S. (2016). Translating person-centered care into practice: A comparative analysis of motivational interviewing, illness-integration support, and guided self-determination. *Patient Education and Counseling*.

[12] Niemiec C. P., Ryan R. M., Deci E. L. (2010). *Self-determination theory and the relation of autonomy to self-regulatory processes and personality development*.

[13] Williams G. C., Deci E. L., Ryan R. M. (2002). Improving Patients Health through Supporting the Autonomy of Patients and Providers. *Handbook of Self-Determination Research*.

[14] Williams G. C., Patrick H., Niemiec C. P. (2009). Reducing the health risks of diabetes: How self-determination theory may help improve medication adherence and quality of life. *The Diabetes Educator*.

[15] Halvari A. E. M., Halvari H., Williams G. C., Deci E. L. (2017). Predicting dental attendance from dental hygienists’ autonomy support and patients’ autonomous motivation: A randomised clinical trial. *Psychology & Health*.

[16] Deci E. L., Ryan R. M. (2000). Self-determination theory and the facilitation of intrinsic motivation, social development, and well-being. *American Psychologist (Salma)*.

[17] Ng J. Y. Y., Ntoumanis N., Thøgersen-Ntoumani C. (2012). Self-Determination Theory Applied to Health Contexts: A Meta-Analysis. *Perspectives on Psychological Science*.

[18] Phillips A. S., Guarnaccia C. A. (2017). Self-determination theory and motivational interviewing interventions for type 2 diabetes prevention and treatment: A systematic review. *Journal of Health Psychology*.

[19] Gourlan M., Trouilloud D., Boiché J. (2016). Motivational Profiles for Physical Activity Practice in Adults with Type 2 Diabetes: A Self-Determination Theory Perspective. *Behavioral Medicine*.

[20] Zoffmann V., Vistisen D., Due-Christensen M. (2015). Flexible guided self-determination intervention for younger adults with poorly controlled Type 1 diabetes, decreased HbA_1c_ and psychosocial distress in women but not in men: a real-life RCT. *Diabetic Medicine*.

[21] Zoffmann V., Lauritzen T. (2006). Guided self-determination improves life skills with Type 1 diabetes and A1C in randomized controlled trial. *Patient Education and Counseling*.

[22] Jørgensen R., Licht R. W., Lysaker P. H. (2015). Effects on cognitive and clinical insight with the use of Guided Self-Determination in outpatients with schizophrenia: A randomized open trial. *European Psychiatry*.

[23] Husted G. R., Esbensen B. A., Hommel E., Thorsteinsson B., Zoffmann V. (2014). Adolescents developing life skills for managing type 1 diabetes: A qualitative, realistic evaluation of a guided self-determination-youth intervention. *Journal of Advanced Nursing*.

[24] Craig P., Dieppe P., Macintyre S., Mitchie S., Nazareth I., Petticrew M. (2008). Developing and evaluating complex interventions: the new Medical Research Council guidance. *British Medical Journal*.

[25] Health T. N. D. o. (2016). *Nasjonal faglig retningslinje for diabetes (in Norwegian)*.

[26] Zoffmann V. (2004). *Guided Self-Determination: a life skills approach in difficult type 1 diabetes*.

[27] Graneheim U. H., Lundman B. (2004). Qualitative content analysis in nursing research: concepts, procedures and measures to achieve trustworthiness. *Nurse Education Today *.

[28] Zoffmann V., Kirkevold M. (2012). Realizing empowerment in difficult diabetes care: A guided self-determination intervention. *Qualitative Health Research*.

[29] Deci E. L., Ryan R. M. (2002). *Handbook of self-determination research*.

[30] Deci E. L., Ryan R. M. (2000). The ‘what’ and ‘why’ of goal pursuits: human needs and the self-determination of behavior. *Psychological Inquiry*.

[31] Cooper H. C., Booth K., Gill G. (2003). Patients' perspectives on diabetes health care education. *Health Education Research*.

[32] Brobeck E., Odencrants S., Bergh H., Hildingh C. (2014). Patients' experiences of lifestyle discussions based on motivational interviewing: A qualitative study. *BMC Nursing*.

[33] Anderson R. M., Funnell M. M. (2010). Patient empowerment: myths and misconceptions. *Patient Education and Counseling*.

[34] Ljung S., Olsson C., Rask M., Lindahl B. (2013). Patient experiences of a theory-based lifestyle-focused group treatment in the prevention of cardiovascular diseases and type 2 diabetes. *International Journal of Behavioral Medicine*.

[35] Funnell M. M., Anderson R. M., Arnold M. S. (1991). Empowerment: an idea whose time has come in diabetes education. *The Diabetes Educator*.

[36] Moser A., van der Bruggen H., Widdershoven G. (2006). Competency in shaping one's life: Autonomy of people with type 2 diabetes mellitus in a nurse-led, shared-care setting; a qualitative study. *International Journal of Nursing Studies*.

[37] Kirk S. F. L., Penney T. L., McHugh T.-L. F., Sharma A. M. (2012). Effective weight management practice: a review of the lifestyle intervention evidence. *International Journal of Obesity*.

[38] Clark M. (2008). Diabetes self-management education: a review of published studies. *Primary Care Diabetes*.

